# Clinical and biochemical assessments of circulating High Mobility Group Box Protein1 in children with epilepsy: relation to cognitive function and drug responsiveness

**DOI:** 10.1007/s10072-024-07795-z

**Published:** 2024-10-28

**Authors:** Hala M. Sakhr, Mohammed H. Hassan, Asmaa E. Salah, Ali Helmi Bakri

**Affiliations:** 1 Department of Pediatrics, Faculty of Medicine, South Valley University, Qena, Egypt; 2 Department of Medical Biochemistry, Faculty of Medicine, South Valley University, Qena, Egypt

**Keywords:** Cognitive function, Drug-resistant epilepsy, High Mobility Group Box Protein1

## Abstract

**Background:**

Childhood epilepsy is a major health concern posing a significant burden and having disastrous consequences for cognitive function. High Mobility Group Box1 (HMGB1) is an activator of neuroinflammation, and it is possibly involved in the initiation and progression of epilepsy. We aimed to investigate circulating HMGB1 in children with epilepsy and its connection to cognitive function and drug responsiveness.

**Methods:**

Case-control research included 100 epileptic youngsters and 100 healthy matched controls. Serum HMGB1 was measured using a commercially available ELISA assay. Cognitive functions were evaluated by the Stanford-Binet test 5th edition.

**Results:**

Drug-resistant epilepsy (DRE) was found in 37% of the investigated patients. Epileptic children have lower cognitive function parameter levels versus the control group and lower cognitive function in the DRE group compared to the drug-responsive group (P-value < 0.0001). HMGB1 levels were significantly higher in the patients’ group (6.279 µg/L) compared to the control group (2.093 µg/L) and in the drug-resistant group (14.26 µg/L) versus the drug-responsive group (4.88 µg/L). A significant negative correlation was detected between HMGB1 with Full-scale IQ (*r* = − 0.547, *P* = 0.000), Visual-spatial reasoning (*r* = − 0.501, *P* = 0.000), fluid reasoning (*r* = − 0.510, *P* = 0.000), and working memory (*r* = − 0.555, *P* = 0.000). Serum HMGB1 cut-off levels > 6.85 µg/L differentiate drug-responsive from resistant patients.

**Conclusion:**

Elevated HMGB1 levels, especially in patients with drug-resistant epilepsy, correlate negatively with cognitive performance, emphasizing its importance as a potential marker for early prediction of drug resistance and impairment of cognitive function.

## Introduction

Seizures are among the most frequent neurological symptoms in children. Globally, almost 11 million children under the age of 15 have active epilepsy [1]. In 2017, more than 291 million children under the age of 20 had epilepsy and intellectual disabilities, with 95% living in low- and middle-income countries [2].

The International League Against Epilepsy defines drug-resistant epilepsy (DRE) as the failure of adequate trials of two well-tolerated, adequately chosen, and used Anti-seizure medication (ASM) schedules (either as monotherapies or combinations) to achieve persistent seizure independence [3]. More than 30% of epileptic patients develop drug-resistant epilepsy [4], and there are increasing reports of adverse consequences such as cognitive impairment, lethargy, and behavioral dysfunction in a considerable proportion of patients treated with ASM persistently [5].

Frequent and severe epileptic seizures are thought to contribute to additional brain injury and long-term neurobehavioral and neuropsychiatric disorders, with devastating consequences for patients, their families, and society [6]. Early detection of DRE is critical for improving patient outcomes. Unfortunately, the process underlying drug resistance is still hypothetical [7, 8]. Therefore, the diagnosis of DRE remains dependent on the outcome of pharmacological treatment [9].

Several studies have revealed that neuroinflammation can cause seizure onset and recurrence by increasing neuronal excitability. Notably, microglia and astrocytes can increase neuroinflammation and seizure susceptibility, where inflammatory mediators generated by glial cells may boost neuronal excitation, resulting in medication resistance and seizure recurrence [10].

Some studies have documented the use of HMGB1 as a biomarker for epilepsy, which can be used to assess illness progression and has also proven to diagnose DRE [11, 12]. HMGB1, a nuclear DNA binding protein, is regarded as a novel pro-inflammatory cytokine. It has been intensively researched because of its role as an alarm protein that triggers innate immunity [13]. HMGB1 is an activator and amplifier of neuroinflammation, and stimulates Toll-like receptor 4 (TLR4) in neurons and astrocytes, resulting in the production of proinflammatory cytokines [14].

The development of biomarkers can aid in predicting patients who are at risk of developing drug resistance, which is critical to the outcome of clinical trials of novel treatments for drug resistance treatment or prevention [15]. Early detection of pediatric patients with drug-resistant epilepsy is critical for appropriate treatment selection and avoiding the devastating consequences of recurrent seizures.

Our hypothesis was that drug-resistant epileptic youngsters who experienced numerous seizures and received multiple ASM would have a negative impact on their cognitive function. Our research question was whether there was a link between HMGB1 in children with epilepsy and their cognitive function and drug responsiveness, so our primary goal was to determine if there is a cognitive function impairment in epileptic patients compared to the control group, and if there is a difference between these cognitive areas in DRE and drug-responsive groups. The secondary purpose was to assess the possible link between HMGB1 levels in these children and its relationship to cognitive function and drug responsiveness.

## Patients and methods

### Study design and participants

According to the Declaration of Helsinki guidelines, a hospital-based case-control study was done to investigate the potential link between the risk of DRE and outcomes on cognitive function, as well as how this exposure differed between cases and controls. The study was conducted with 200 children aged 2–16 years old; 100 children diagnosed with epilepsy [16] who were attending the Pediatric Neurology Clinic and Pediatric Department during the studying period from January 2023 to December 2023, who were compared with 100 healthy-matched control groups attended the pediatric clinic for routine growth monitoring and were unrelated to the included cases.

Exclusion criteria included patients with cerebral palsy, mental or psychiatric illnesses, metabolic abnormalities, concomitant chronic disease, cases with previous traumatic brain injury, syndromic epilepsy, and patients with any abnormalities in CT or MRI brain, in an attempt to control the confounders of the study.

### Ethical consideration

Approval of the ethics committee of the University was obtained (Ethical approval code: SVUMEDPED0254245849), and all participants’ caregivers provided informed written consent.

### Clinical evaluation of the participants

All participants provided primary information, such as their age, gender, and residence. Clinical data on the disease history were collected, including family history of epilepsy, age of the first seizure attack, type of seizure onset [17], duration of fits, frequency of attacks in the last two years of therapy, prior history of status epilepticus, and accompanying symptoms. Pharmacotherapy details included the type of antiepileptic medicine used, the dose, and the patient’s response to therapy. Epileptic children were divided into two groups according to their drug responsiveness [3]. Every subject underwent a complete general and systematic examination and Body mass index (BMI) was calculated (kg/m2).

### Cognitive function assessment

the Arabic version of the Stanford-Binet test 5th edition, was used to evaluate Full-Scale Intelligence Quotient (IQ), Working memory, Quantitative reasoning, Fluid Reasoning, Visual-Spatial Reasoning, and Knowledge. Cognitive domains are classed according to the range as highly advanced (140+), very advanced (130–140), superior (120–129), high average (110–119), average (90–109), low average (80–89), borderline impaired (70–79), mildly impaired (55–69), and moderately impaired (40–54) [18].

### High Mobility Group Box Protein1 measurements

To avoid the acute response that occurs after a seizure, blood samples were collected at least 24 h after seizure activity [19, 20] as follows; 2 ml of venous blood was withdrawn from each participant on an EDTA tube, then centrifuged at 3500 rpm at 37° for 10 min, then the separated plasma was aliquoted and stored into 1 mL cryotubes, and stored at -80° until the High Mobility Group Box Protein1 measurements by commercially available ELISA kits (Chongqing Biospes Co., Ltd., China provided with catalog number BZEK1738-48), using microplate ELISA reader EMR 500, USA.

### Interictal electroencephalographic (EEG) recording

EEG recording with a Nihon Khoden 8-channel conventional EEG machine (NIHON KOHDENMEB-9400) was used as previously mentioned by our previous studies [21, 22].

## Statistical analyses

Statistical analysis was carried out using Statistical Programme for the Social Sciences software version 24. Mann-Whitney tests were used to compare the two groups in a non-normally distributed continuous dataset and Spearman’s rank correlation was used to examine the relationship between non-normally distributed continuous variables. Chi-square test was used to compare differences between Categorical variables. The Receiver Operating Characteristic Curve (ROC curve) was used to determine cutoff values, sensitivity, specificity, positive predictive value (PPV), and negative predictive value (NPV). Statistical significance was defined as a P-value < 0.05.

## Results

**Fig. 1:**
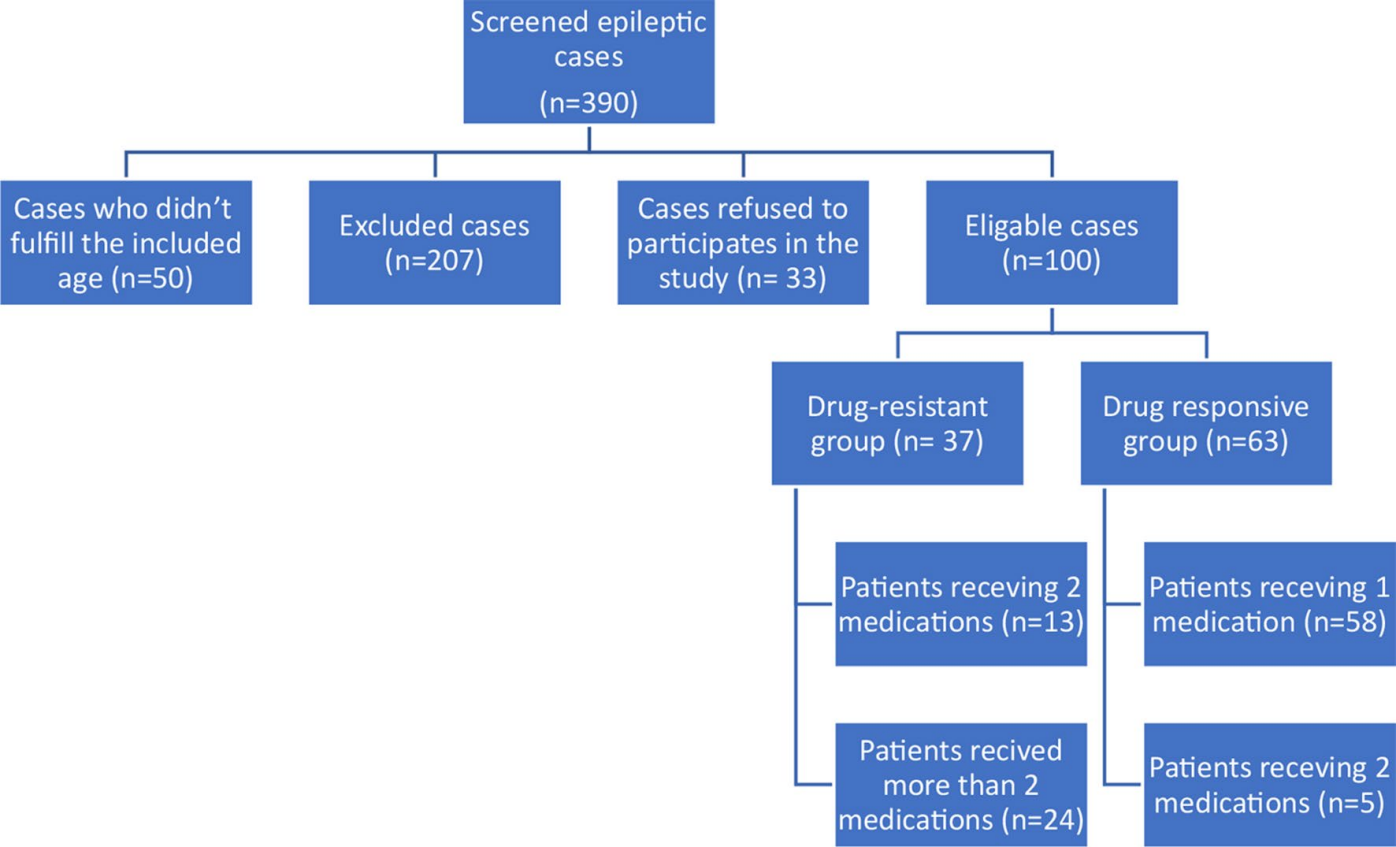
Flowchart of the screened epileptic patients

Out of 390 children diagnosed with epilepsy, 100 eligible children (Fig. 1); 46 boys and 54 girls with a median age of 6 years were compared to 100 matched healthy participants with no significant differences between the two groups as regards age, gender, domicile, parental consanguinity, or body mass index. Family history of epilepsy was significant in epileptic group (47% in epileptic children versus 12% in the healthy group) Table 1.

**Table 1:** Descriptive data of the studied participants

	Epileptic patients (*N* = 100)	Healthy children (*N* = 100)	*P*. Value
Age (years) (Median – IQR)	6.0 (4.0–9.4)	6.0 (6–6.5)	0.182
Sex (N%)			
Male Female	46 (46%) 54 (54%)	40 (40%) 60 (60%)	0.485
Residence (N%)			
Rural Urban	50 (50%) 50 (50%)	52 (52%) 48 (48%)	0.817
Parental consanguinity(N%)	48 (48%)	36 (36%)	0.163
Family history of epilepsy(N%)	47 (47%)	12 (12%)	< 0.0001**
BMI (kg/m2)	15.59 (15.0–17.5)	15.8 (15.6–16)	0.292

Cm: centimeter. – IQR: interquartile range. - Kg: Kilograms. * Statistically significant predictor (*p* < 0.05). ** Highly statistically significant predictor (*p* < 0.001)

Epileptic cases had a median and IQR of Full-scale IQ of 90 (82-110.5), which was considerably lower than the control group’s 103 (97–113), with 27% and 20% of epileptic children having a low average IQ and borderline impaired respectively, whereas only 4% of the control group had low average IQ. The mean levels of working memory, quantitative reasoning, fluid reasoning, visual-spatial reasoning, and knowledge were all significantly affected as compared to the control group, with 12%, 29%, 20%, 29%, and 22% having cognitive impairment respectively in the patient group, as shown in Table 2.

**Table 2:** Intelligence test parameters in patients and control groups

Variable		Epileptic patients (*N* = 100)	Healthy children (*N* = 100)	*P*-value
**Full-scale IQ**	**Median (IQR)**	90 (82- 110.5)	103 (97–113)	< 0.0001 **
**Full-scale IQ** **(N.%)**	**Superior**	10 (10%)	12 (12%)	< 0.0001 **
	**High average**	15 (15%)	34 (34%)	
	**Average**	28 (28%)	50 (50%)	
	**Low average**	27 (27%)	4 (4%)	
	**Borderline impaired**	20 (20%)	0 (0%)	
**Working memory**	**Median (IQR)**	96 (86–106)	101 (95.5–110)	0.005**
**Working memory** **(N.%)**	**Superior**	12 (12%)	12(12%)	< 0.0001 **
	**High average**	11 (11%)	16(16%)	
	**Average**	40 (40%)	68 (68%)	
	**Low average**	25 (25%)	4 (4%)	
	**Borderline impaired**	9 (9%)	0 (0.0%)	
	**Mildly impaired**	3 (3%)	0 (0.0%)	
**Quantitative reasoning**	**Median (IQR)**	88.5 (75–100)	105.5 (92–116)	< 0.0001 **
**Quantitative reasoning** **(N.%)**	**Superior**	6 (6%)	18 (18%)	< 0.0001 **
	**High average**	11 (11%)	20 (20%)	
	**Average**	31 (31%)	62(62%)	
	**Low average**	23 (23%)	0 (0.0%)	
	**Borderline impaired**	29 (29%)	0 (0.0%)	
**Fluid Reasoning**	**Median (IQR)**	94 (82–105)	103 (95–113)	< 0.0001 **
**Fluid Reasoning** **(N.%)**	**Superior**	6 (6%)	18 (18%)	< 0.0001 **
	**High average**	15 (15%)	18 (18%)	
	**Average**	37 (37%)	64 (64%)	
	**Low average**	22 (22%)	0 (0.0%)	
	**Borderline impaired**	20 (20%)	0 (0.0%)	
**Visual-Spatial Reasoning**	**Median (IQR)**	88 (78–104)	100 (92–110)	< 0.0001 **
**Visual-Spatial Reasoning** **(N.%)**	**Superior**	4 (4%)	4 (4%)	< 0.0001 **
	**High average**	17 (17%)	26 (26%)	
	**Average**	23 (23%)	70 (70%)	
	**Low average**	27 (27%)	0 (0.0%)	
	**Borderline impaired**	29 (29%)	0 (0.0%)	
**Knowledge**	**Median (IQR)**	90 (80–100)	100 (95–114)	< 0.0001 **
**Knowledge** **(N.%)**	**Superior**	4 (4%)	10 (10%)	< 0.0001 **
	**High average**	9 (9%)	20 (20%)	
	**Average**	41 (41%)	62 (62%)	
	**Low average**	24 (24%)	8 (8%)	
	**Borderline impaired**	22 (22%)	0 (0.0%)	

IQ: Intelligence Quotient. ** Highly statistically significant predictor (*p* < 0.001)

Drug-resistant epilepsy was found in 37% of the cases investigated, with a higher median age of 7 years (IQR of 5–12 years) than children in the drug-responsive group (median age of 5.5 years and IQR of 4–6.5 years). The majority of cases in the drug-responsive group (92.1%) were controlled with a single medication, while in the DRE group, 35.1% were using two medications and 64.9% were on more than two medications. No significant differences between the groups in terms of sex, residency, family history, parental consanguinity, age at onset, kind of seizure, or duration of fits, while a significant difference was detected as regards previous history of febrile seizures, status epileptics, and EEG abnormalities (Table 3).

**Table 3:** Data comparison between drug-responsive and drug-resistant groups

Variables Median (IQR)	Drug-responsive group (*N* = 63)	Drug-resistant group (*N* = 37)	*P*. Value
**Age (years)**	5.5 (4-6.5)	7 (5–12)	< 0.0001 **
**Sex**:			
Males Females	33 (52.4%) 30 (47.6%)	13 (35.1%) 24 (64.9%)	0.095
**Residence**:			
Rural Urban	31 (49.2%) 32 (50.8%)	19 (51.4%) 18 (48.6%)	0.836
**Family History**	30 (47.6%)	17 (45.9%)	0.871
**Parental consanguinity**	28(44.4%)	19 (51.4%)	0.504
**Age at onset (years)**	2 (1.5–4)	3.2 (2.5–5)	0. 121
**Type of Seizure onset**:			
Generalized onset Focal onset	50 (79.4%) 13 (20.6%)	33 (89.2%) 4 (10.8%)	0.206
**Duration of fits (min)**	7.5 (4–15)	7.5 (3.5–13.75)	0.626
**Frequency of attacks in last 2 years)**	2 (2–4)	28 (25–45)	< 0.0001**
**Febrile seizures**:			
yes No	43 (68.3%) 20 (31.7%)	17 (45.9%) 20 (54.1%)	0.027*
**History of status epilepticus**:			
None Once Frequent	53 (84.1%) 10 (15.9%) 0 (0.0%)	14 (37.83%) 10 (27.02%) 13 (35.13%)	< 0.0001**
**Number of medications**:			
One medication Two medications > two medications	58 (92.1%) 5 (7.9%) 0 (0.0%)	0 (0.0%) 13 (35.1%) 24 (64.9%)	< 0.0001**
**BMI (kg/m2)**	15.7 (14.2–16.7)	16.5 (15.25–17.65)	0.023*
**Interictal EEG Findings**:			
Focal Epileptic Discharge Generalized Epileptic Discharge Cerebral dysrhythmia Normal EEG	5 (7.9%) 32 (50.8%) 10(15.9%) 16 (25.4%)	8 (21.6%) 21 (56.8%) 6(16.2%) 2 (5.4%)	0.033*

BMI: Body mass index. - EEG: Electroencephalogram. * Statistically significant predictor (*p* < 0.05). ** Highly statistically significant predictor (*p* < 0.001)

Children with DRE had a significantly lower full-scale IQ of 76 (72.5–84) compared to 97 (90–116) in the drug-responsive group. Other cognitive function measures were also considerably lower in the drug-resistant group versus the drug-responsive group. Cognitive impairment in the drug-resistant group was detected in 54.1%, 27%, 78.4%, and 51.4%, 62.2%, and 54.1% in Full-scale IQ, working memory, quantitative reasoning, fluid reasoning, visual-spatial reasoning, and knowledge respectively versus 0.0%, 3.2%,0.0%, 1.6%, 9.5%, 3.2% in the drug-responsive group (Table 4).

**Table 4:** Intelligence test parameters in patients’ subgroups

Variable		Drug-responsive group (*N* = 63)	Drug-resistant group (*N* = 37)	*P*-value
**Full-scale IQ**	**Median (IQR)**	97 (90–116)	76 (72.5–84)	< 0.0001 **
**Full-scale IQ** **(N.%)**	**Superior**	10 (15.9%)	0 (0%)	< 0.0001 **
	**High average**	15 (23.8%)	0 (0%)	
	**Average**	23(36.5%)	5 (13.5%)	
	**Low average**	15 (23.8%)	12 (32.4%)	
	**Borderline impaired**	0 (0%)	20 (54.1%)	
**Working memory**	**Median (IQR)**	100 (97–118)	86 (78–89)	< 0.0001 **
**Working memory** **(N.%)**	**Superior**	12 (19%)	0 (0.0%)	< 0.0001 **
	**High average**	11 (17.5%)	0 (0.0%)	
	**Average**	31 (49.2%)	9 (24.3%)	
	**Low average**	7 (11.1%)	18 (48.6%)	
	**Borderline impaired**	2 (3.2%)	7 (19%)	
	**Mildly impaired**	0(0.0%)	3 (8.1%)	
**Quantitative reasoning**	**Median (IQR)**	97 (89–113)	75 (72–75)	< 0.0001 **
**Quantitative reasoning** **(N.%)**	**Superior**	6 (9.5%)	0(0.0%)	< 0.0001 **
	**High average**	11 (17.5%)	0(0.0%)	
	**Average**	29 (46%)	2 (5.4%)	
	**Low average**	17 (27%)	6 (16.2%)	
	**Borderline impaired**	0(0.0%)	29 (78.4%)	
**Fluid Reasoning**	**Median (IQR)**	100 (90–115)	79 (74.5–94)	< 0.0001 **
**Fluid Reasoning** **(N.%)**	**Superior**	6 (9.5%)	0 (0.0%)	< 0.0001 **
	**High average**	15 (23.8%)	0 (0.0%)	
	**Average**	27 (42.9%)	10 (27%)	
	**Low average**	14 (22.2%)	8 (21.6%)	
	**Borderline impaired**	1 (1.6%)	19 (51.4%)	
**Visual-Spatial reasoning**	**Median (IQR)**	96 (86–110)	75 (74–88)	< 0.0001 **
**Visual-Spatial Reasoning** **(N.%)**	**Superior**	4 (6.3%)	0 (0.0%)	< 0.0001 **
	**High average**	17 (27%)	0 (0.0%)	
	**Average**	19 (30.2%)	4 (10.8%)	
	**Low average**	17 (27%)	10 (27%)	
	**Borderline impaired**	6 (9.5%)	23(62.2%)	
**Knowledge**	**Median (IQR)**	98 (88–108)	78 (72–85)	< 0.0001 **
**Knowledge** **(N.%)**	**Superior**	4 (6.3%)	0 (0.0%)	< 0.0001 **
	**High average**	9 (14.3%)	0 (0.0%)	
	**Average**	33 (52.4%)	8 (21.6%)	
	**Low average**	15 (23.8%)	9 (24.3%)	
	**Borderline impaired**	2 (3.2%)	20 (54.1%)	

IQ: Intelligence Quotient. ** Highly statistically significant predictor (*p* < 0.001)

Serum HMGB1 levels were considerably higher in the patients’ group, with median levels of 6.047 µg/L (4.65–11.05) compared to 2.093 µg/L (1.41–3.25) in the healthy group, additionally, the drug-resistant group had a substantially higher median blood level of HMGB1 (14.26 µg/L, IQR 9.30–14.26) compared to the drug-responsive group’s (4.88 µg/L, IQR 4.65–5.12)(Table 5).

**Table 5:** Serum level of High Mobility Group Box Protein1 in the studied participants subgroups

Median (IQR)	Serum HMGB1(µg/L)	*P*. Value
**Epileptic patients versus healthy controls**		
Epileptic patients (*N* = 100)	6.047 (4.65–11.05)	< 0.0001 **
Healthy children (*N* = 100)	2.093 (1.41–3.25)	
**Drug-responsive versus Drug-resistant group**		
Drug-responsive group (*N* = 63)	4.88 (4.65–5.12)	< 0.0001 **
Drug-resistant group (*N* = 37)	14.26 (9.30–14.26)	

* Statistically significant predictor (*p* < 0.05). ** Highly statistically significant predictor (*p* < 0.001). -HMGB1: High Mobility Group Box Protein1

**Fig. 2:**
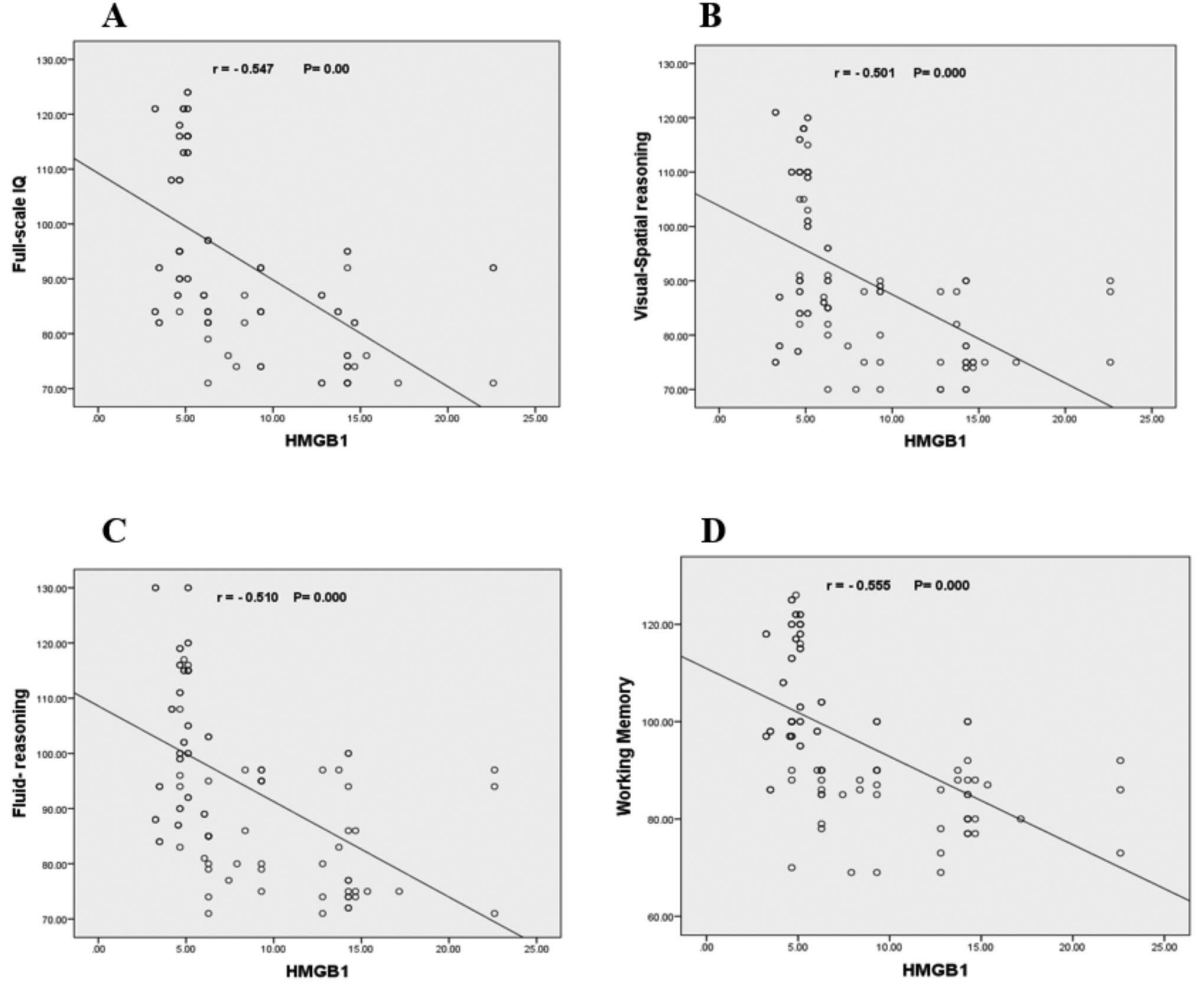
Correlation of High Mobility Group Box Protein1 with Full-scale IQ (**A**), Visual-spatial reasoning (**B**), Fluid-reasoning (**C**), and Working memory (**D**) in epileptic patients

**Fig. 3:**
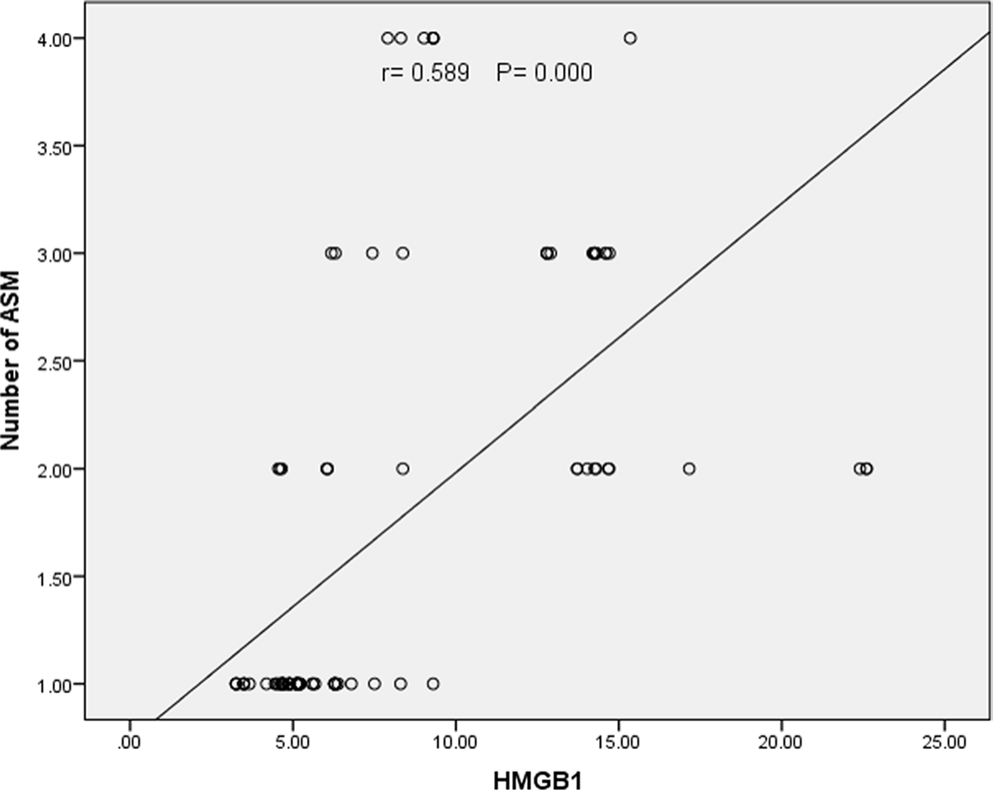
Correlation of High Mobility Group Box Protein1 with the number of anti-seizure medications in epileptic patients

Figures 2 A, B, C, and D indicate a significant negative connection between HMGB1 and full-scale IQ (*r* = − 0.547, *P* = 0.000), visual-spatial reasoning (*r* = − 0.501, *P* = 0.000), fluid reasoning (*r* = − 0.510, *P* = 0.000), and working memory (*r* = − 0.555, *P* = 0.000). Fig 3 demonstrates a strong positive correlation between HMGB1 and the number of ASM (*r* = 0.589, *P* = 0.000)

**Fig. 4:**
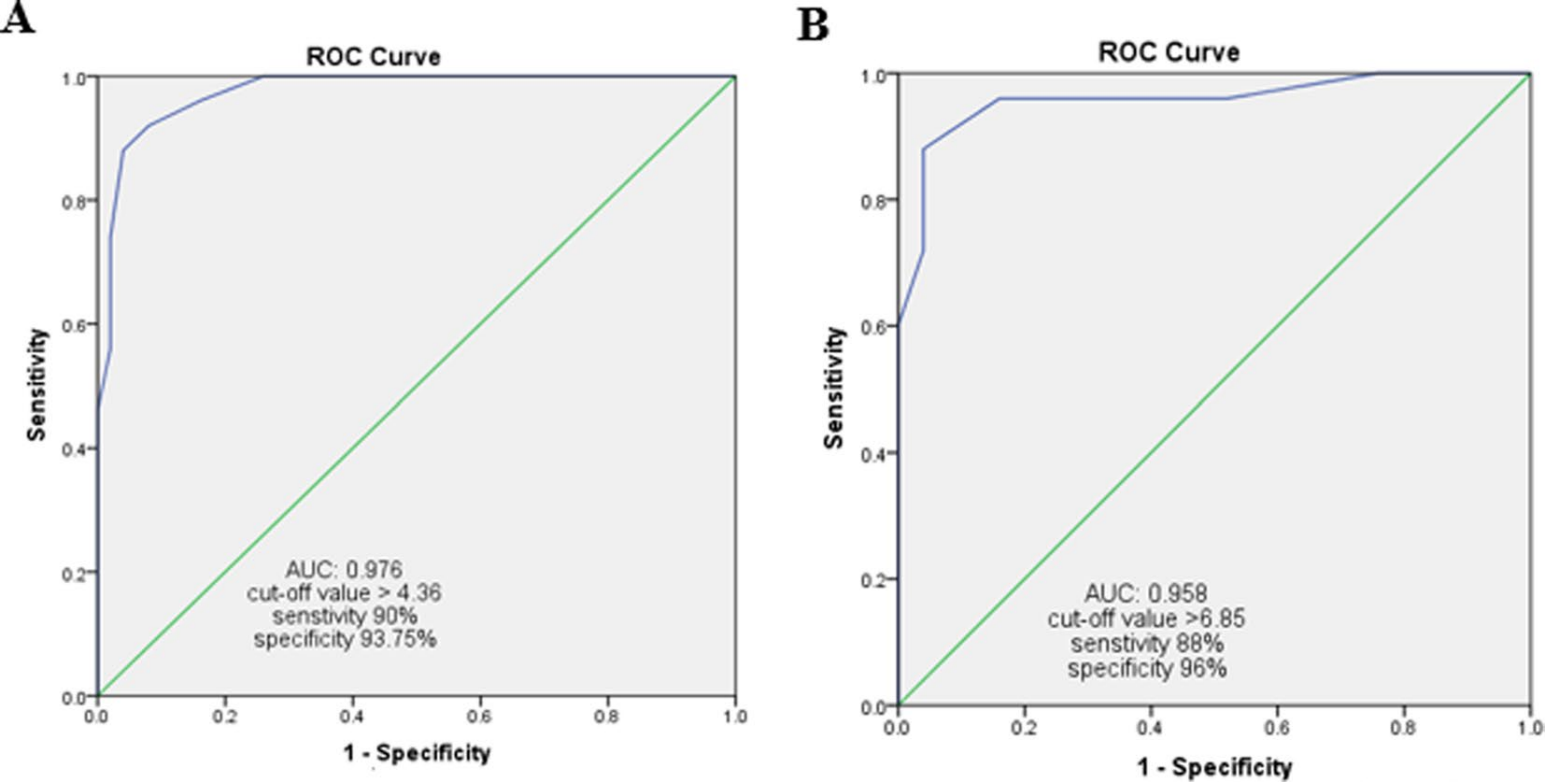
ROC curve analysis of High Mobility Group Box Protein1 in discriminating cases versus control groups (**A**) and Drug-responsive versus Drug-resistant (**B**)

Using a cutoff value of > 4.36 µg/L, serum HMGB1 exhibited a significant rise in area under the curve (AUC) to 0.976, indicating high discriminatory power between cases and the control group. Notably, at this cutoff, sensitivity reached 90%, specificity was 94%, PPV was 93.75%, and NPV was 90.4%. Using a threshold of > 6.85 µg/L, serum HMGB1 can differentiate between patient subgroups with an AUC of 0.958, sensitivity was 88%, specificity was 96%, PPV of 95.6%, and NPV of 88.9%. Fig 4 A & B.

## Discussion

Epilepsy is a major health concern and continues to have a large illness burden in children and adolescents in Africa [23]. Most epileptic patients can expect satisfactory seizure control with anti-seizure medications. Nonetheless, around 20–40% of children with epilepsy experience refractory episodes despite effective treatment [24, 25]. In the current research drug-resistant epilepsy was found in 37% of the investigated patients which also concedes with Chen et al. [4] results.

Patients with DRE have poor medication responsiveness and greater seizure frequency. Cognitive impairment is one of the most common and devastating aspects of epilepsy [26, 27]. We found that cognitive function domains were highly affected in epileptic children and significantly lower in comparison to the matched control group and also it is more affected in the drug-resistant group when compared to the drug-responsive group.

Lee et al. [28] found a mean IQ of 92.8 ± 16.5 in their study of epileptic children and Mohamed et al. [29] found that 47.1% of their patients had an average Full-Scale IQ score, 23.5% had a low average, and 9.6% had borderline impaired or delayed scores. Given that the temporal lobes and hippocampus are connected with memory formation, it is not surprising that seizures in these areas might produce memory problems. Neurotransmitters play a crucial role in sustaining normal brain processes and are known to be altered during a brain insult. Alterations in neurotransmitters have been linked to epilepsy. GABA, glutamate, and acetylcholine are three essential neurotransmitters that have been linked to epilepsy and cognition [30]. Kundap et al. [31] showed that in an animal investigation, all antiseizure medications have a negative influence on cognitive abilities.

In line with our findings, several investigations found that epilepsy patients had greater HMGB1 concentrations than the control group [32–34], and as an independent risk factor for epilepsy prognosis [35]. While others also indicated higher levels in treatment-resistant epilepsy than drug responsiveness or control group [36–40].

Over the past decade, many studies have highlighted the crucial pathophysiological role of brain inflammation in epilepsy [41–43]. The effects of HMGB1 on many neurological illnesses are quite intriguing. It is indisputable that normal intracellular HMGB1 can be involved in repair and autophagy regulation, whereas extracellular HMGB1 initiates and amplifies neuroinflammation, and the onset of a neuroinflammatory response can be attributed to intrinsic “harmful” events. In neuroinflammation, activated neurons and microglia cells express HMGB1, which increases the release of inflammatory molecules such as TNF-α, IL-1β, and IL-6. This activation of associated pathways leads to neurological damage and dysfunction [44]. Maroso et al. [45] also discovered a pro-convulsant pathway involving HMGB1 release from neurons and glia and its interaction with TLR4, a critical receptor of innate immunity. They reported that antagonists of HMGB1 and TLR4 slow seizure onset and reduce acute and chronic seizure occurrence.

HMGB1 is typically released from neurons and glial cells during and after seizures as part of the cellular stress response. Serum HMGB1 levels tend to peak within a few hours post-seizure following a seizure event, depending on the intensity and duration of the seizure [19]. In some studies, elevated HMGB1 levels have been observed up to 24 h after a seizure [20]. While in patients with chronic epilepsy, baseline levels of HMGB1 may remain elevated. Pekny et al., [46] highlight how chronic neuroinflammation in epilepsy can lead to persistently high HMGB1 levels, affecting the dynamics of its response to acute seizure events. This persistent elevation may influence both the severity of seizures and the long-term outcomes in individuals with epilepsy.

we demonstrated a strong positive correlation between HMGB1 and the number of ASM. The causes of cognitive dysfunction in DRE may be multifocal, with a complex interaction of multiple factors such as frequent and severe epileptic seizures and the use of multiple ASM.

Multiple studies have investigated the influence of ASM on cognitive performance, with conflicting results. Foster et al. [47] stated that no ASM was independently associated with cognitive dysfunction beyond other clinically relevant factors. Shafyev and Karadaş [48] found that certain ASMs, particularly topiramate and carbamazepine, have a negative impact on cognitive processes. Furthermore, the deleterious impact on cognitive performance became more evident with a growing number of concurrently utilized ASMs (polytherapy). However, they highlighted that one limitation of their study is that patients receiving polytherapy may be at risk for treatment-resistant epilepsy, which could impair cognition over long durations of multiple drug use.

We figured out a significant negative correlation between HMGB1 with Full-scale IQ, Visual-spatial reasoning, fluid reasoning, and working memory. In concordance with our findings, Zhu et al., [33] documented that serum concentrations of HMGB1 were negatively associated with patients’ intelligence scores and Huang et al. [34] discovered that the expression levels of HMGB1 in the peripheral blood of epileptic patients are significantly higher and adversely linked with neurological function scores.

HMGB1 is generated after tissue damage, recurrent seizures, and inflammation and remains increased long after a severe systemic inflammatory insult, causing hippocampal inflammation and cognitive impairment in experimental mice [45, 49–51]. Interestingly, anti-HMGB1 medication provided many days after severe sepsis can reduce cognitive impairment in mice [49]. Ganai and Husain [52], in their study on a rat model with hepatic encephalopathy, have reported that cognitive impairment has been ameliorated via alleviating neuroinflammation. Tan et al., [53] also in an animal study discovered that the NOD-, LRR-, and pyrin domain-containing protein 3 (NLRP3) inflammasome harms memory in the late stages of traumatic brain injury, predominantly through HMGB1 overexpression, which explains the long-term progression of cognitive loss. Yin et al. [54] discovered that HMGB1 causes microglial activation, abnormal synaptic pruning, and neuron malfunction in an animal model of sepsis-associated encephalopathy, resulting in cognitive impairment.

Mazarati et al. [55] have shown in an experimental investigation of mice that higher brain levels of HMGB1 cause memory impairments that may be mediated by TLR4 or Receptor for Advanced Glycation End Products (RAGE). This pathway may contribute to memory problems in a variety of neurological and psychiatric diseases characterized by elevated HMGB1 levels, including epilepsy, Alzheimer’s disease, and stroke.

Zhao et al. [56] found that the injection of anti-HMGB1 monoclonal antibodies in mice delayed epilepsy development and improved cognitive skills. The precise mechanism of HMGB1’s participation in cognitive decline is still limited, and more research is needed.

### Limitations of the study

Our study’s weakness is its limited sample size, which is due to its single-center design. A multicenter study should be organized to generalize the current study’s findings with more longitudinal research to better understand ASM role, effects of early drug resistance detection, and HMGB role, which will provide critical insight into how these variables influence cognitive function and affect patients’ prognoses.

## Conclusion

Drug-resistant epilepsy was discovered in 37% of the patients studied, who had a higher frequency of episodes, a history of status epilepticus, and a higher prevalence of aberrant EEG results. Drug-resistant epilepsy (DRE) is a major concern, posing a significant burden and having disastrous repercussions for cognitive function. Elevated HMGB1 levels, particularly in patients with drug-resistant epilepsy, correlate negatively with cognitive performance while correlating positively with the number of ASM, emphasizing its significance as a potential marker for early prediction of drug resistance and impairment of cognitive function, potentially improving long-term outcomes.

## Data Availability

The datasets used and/or analyzed during the current study are available from the corresponding author upon reasonable request, after obtaining the permission of our institute.
